# Emergence and structure of decentralised trade networks around dark web marketplaces

**DOI:** 10.1038/s41598-022-07492-x

**Published:** 2022-03-31

**Authors:** Matthieu Nadini, Alberto Bracci, Abeer ElBahrawy, Philip Gradwell, Alexander Teytelboym, Andrea Baronchelli

**Affiliations:** 1 Department of Mathematics, City University of London, London, EC1V 0HB UK; 2 The Alan Turing Institute, British Library, 96 Euston Road, NW1 2DB London, UK; 3 Chainalysis Inc, New York, NY USA; 4 Department of Economics and INET Oxford, University of Oxford, Oxford, OX1 3UQ UK; 5 UCL Centre for Blockchain Technologies, University College London, London, UK

**Keywords:** Applied physics, Computational science, Statistical physics, thermodynamics and nonlinear dynamics

## Abstract

Dark web marketplaces (DWMs) are online platforms that facilitate illicit trade among millions of users generating billions of dollars in annual revenue. Recently, two interview-based studies have suggested that DWMs may also promote the emergence of direct user-to-user (U2U) trading relationships. Here, we carefully investigate and quantify the scale of U2U trading around DWMs by analysing 31 million Bitcoin transactions among users of 40 DWMs between June 2011 and Jan 2021. We find that half of the DWM users trade through U2U pairs generating a total trading volume greater than DWMs themselves. We then show that hundreds of thousands of DWM users form *stable* trading pairs that are persistent over time. Users in such stable pairs turn out to be the ones with the largest trading volume on DWMs. Then, we show that new U2U pairs often form while both users are active on the same DWM, suggesting the marketplace may serve as a catalyst for new direct trading relationships. Finally, we reveal that stable U2U pairs tend to survive DWM closures and that they were not affected by COVID-19, indicating that their trading activity is resilient to external shocks. Our work unveils sophisticated patterns of trade emerging in the dark web and highlights the importance of investigating user behaviour beyond the immediate buyer-seller network on a single marketplace.

## Introduction

Since the launch of Silk Road, the first modern dark web marketplace (DWM), in 2011^1^ millions of buyers and sellers have traded on the dark web. DWMs have gained popularity because their users can easily and anonymously access them through browsers, such as The Onion Router (Tor)^2^, and trade goods using cryptocurrencies, such as Bitcoin^3^. They offer a variety of illicit goods including drugs, firearms, credit cards dumps, and fake IDs^4^. Indeed, DWMs could represent a threat to society and public health. For instance, during the COVID-19 pandemic, DWMs sold COVID-19 related goods (e.g., masks and COVID-19 tests) that were in shortage in regulated marketplaces as well as unapproved vaccines and fake treatments^5–7^. Law enforcement agencies have therefore targeted DWMs and users trading on them, performing dozens of arrests and seizing millions of US dollars worth of Bitcoin^8–10^. Despite police raids and unexpected closures, DWM trading volume has been steadily increasing and exceeded $1.5 billion for the first time in 2020^11^.

DWM users display complex trading patterns within the marketplace environment. For example, users migrate to alternative DWMs when a DWM that they trade on closes^12,13^. Such migration of users is aided by communication via online forums and chats on the dark web^14,15^. However, little is known about how DWM users trade and transact *outside* the DWMs. On the one hand, some recent works have shown that a significant number of DWM users trade drugs and other illicit goods using social media platforms, such as Facebook, Telegram, and Reddit^16–20^. Moreover, several qualitative, interview-based studies have shown that DWM users form direct trading relationships with other users, starting user-to-user (U2U) pairs that bypass the intermediary role of DWMs^21,22^. Past research has also found that sellers on regulated online marketplaces and social medial platforms may decide to use intermediaries, such as Facebook groups or Instagram, to find new customers, and may start direct U2U trading with potential buyers^23^. In this paper, we look closely at patterns of U2U trading relationships among DWM users.

**Figure 1:**
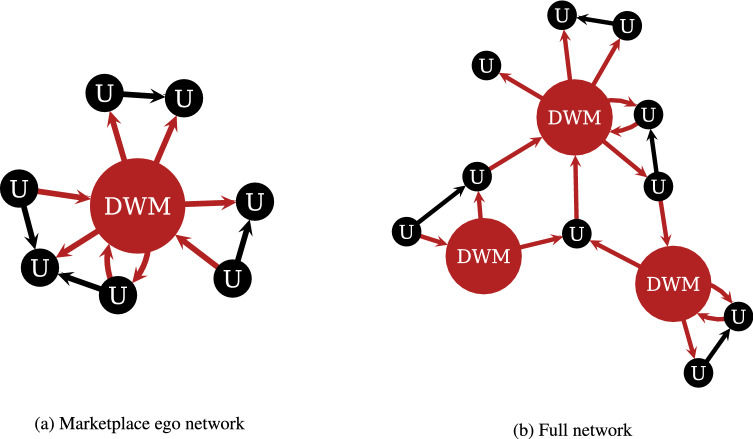
Ego and full networks. (**a**) Schematic representation of an ego network surrounding a dark web marketplace (“DWM”, in red). The DWM interacts with its users (“U”, in black). User-to-user (U2U) pairs are represented by arrows (direction indicates the flow of Bitcoin) and by their respective users. (**b**) Multiple ego networks may be aggregated to form the full network.

The starting point for this paper is the identification of U2U networks around DWMs. We analyse 40 DWMs for the time period spanning from June 18, 2011 to January 31, 2021. Our dataset covers all major DWMs that have ever existed, as identified by the European Monitoring Centre, Europol, the World Health Organization, and independent researchers^24–26^. Our analysis focuses on Bitcoin – the most popular cryptocurrency both on DWMs^27,28^ and in the regulated economy^29,30^. We focus on two kinds of transactions, occurring (i) between the user and a DWM and (ii) between two users of the same DWM. The result is 40 distinct marketplace ego networks containing user-DWM and U2U transactions, whose typical structure is depicted in Fig. 1a. In each network, links are directed and the arrows point at the receiver of Bitcoin. Since users often migrate from one DWM to another^12^ and become users of multiple DWMs, the 40 ego networks are not isolated but can be combined to form one full network, as shown in Fig. 1b.

Previous analyses of U2U trading relationships around DWMs include only two studies^21,22^ based on unstructured^21^ or semi-structured^22^ interviews of 17 users of Silk Road and 13 sellers on various DWMs, respectively. Here, we dramatically extend previous work by exploring the collective emergence and structure of U2U pairs. First, we observe that the U2U network, formed by all transactions between pairs of users, has a larger trading volume than DWMs themselves. We then identify *stable* U2U trading relationships, which represent a subset of persistent pairs in our dataset^31,32^ forming the *backbone* of the U2U network. We find that 137,667 (i.e., 1.7% out of 7.85 million total) pairs are stable, generating a total trading volume of $1.5 billion (i.e., 5% out of $30 billion total volume). We then explore the behaviour of users forming stable U2U pairs. We reveal that stable U2U pairs play a crucial role for marketplaces by spending significantly more time and generating far greater transaction volume with DWMs than other users. By analysing the temporal evolution of stable pairs, we unveil that DWMs acted as meeting points for 37,192 users (out of around 16 million), whose trading volume is estimated to be $417 million. Importantly, these newly formed stable pairs persist over time and transact for several months even after the closure of the DWM that spurred their formation. Finally, we observe that COVID-19 only had a temporary impact on the evolution of stable U2U pairs, which continued to increase their trading volume throughout 2020.

## Results

### Large number of U2U transactions

#### Ego networks

**Figure 2:**
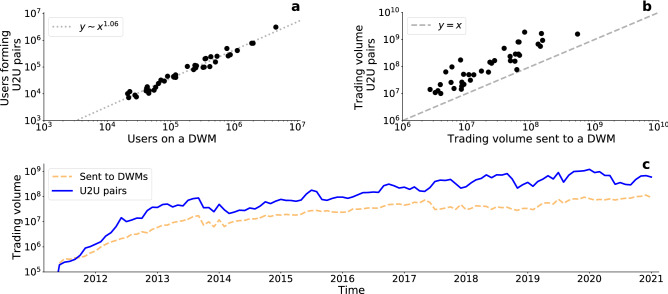
User-DWM and U2U transactions. (**a**) Total number of users interacting with a DWM against the total number of users forming U2U transactions. The dotted line corresponds to the result of a fitted power law function. (**b**) Trading volume in dollars sent to a DWM compared with the total trading volume in its surrounding U2U transactions. The dashed line is the bisector and allows to easily compare the two trading volumes. (**c**) Total monthly trading volume sent to all DWMs and exchanged in all unique U2U pairs. We do not include the trading volume received from DWMs because it is essentially equivalent to the volume sent to DWMs.

We start our analysis by measuring the extent of the U2U network around each DWM. The percentages of users forming U2U pairs vary across DWMs, with a median value of 38% (min 23%, max 68%). The variance in the percentage of users with U2U pairs is shown in Fig. 2a. The Figure shows that the number of users with U2U pairs is almost monomial in the number of users interacting with a DWM, with an estimated exponent equal to 1.06 and $R^2 = 0.969$ , see Section S1 for details on the fitting procedure. The total trading volume users sent to the marketplace is essentially equivalent to the one they receive from it (two-sided Wilcoxon test^33^: $W=330$, $p=0.282$). Importantly, the total trading volume users sent to a DWM (and consequently the one that they receive from it) is always lower than the volume exchanged through U2U transactions, as shown in Fig. 2b.

#### Full network

Similar results hold for the full network, confirming that the formation of U2U pairs is a pervasive phenomenon around DWMs. The total trading volume users sent to DWMs was $3.8 billion, volume received from DWMs was $3.7 billion, while the volume exchanged through U2U pairs reached $30 billion. In Figure S3, we illustrate the number of transactions, trading volume, and lifespan of U2U pairs. In all cases we observe familiar fat-tailed distributions.

We then consider the temporal evolution of transactions. We look at the trading volume over time in Fig. 2c which shows that since 2011 U2U transactions have consistently involved greater monthly volume than the volume sent to all DWMs. This underlines the economic importance of U2U transactions in the Bitcoin ecosystem relative to DWMs.

### Behaviour of the U2U network

Henceforth, we are going to analyse users by focusing on the following groups: users who do not form stable U2U pairs; users who form stable U2U pairs, of which there are users who *met outside* DWMs and users who *met inside* DWMs (see the nomenclature in Table 2). We start by focusing our attention on identifying stable U2U pairs, i.e., persistent pairs of the U2U network. To this end, we use the evolving activity-driven model^31^ to identify stable pairs in a statistically-principled way (see Methods). We find 137,667 stable U2U pairs were formed by 106,648 users and generated a trading volume of $1.5 billion. Stable pairs produce five times more transactions per pair than non-stable pairs (two-sided Mann-Whitney-U test^34^: MNU $$=45.8 \cdot 10^9$$, $$p<0.0001$$) corresponding to a 5.34 times larger trading volume (MNU $$= 317 \cdot 10^9$$, $$p<0.0001$$), see Figure S4. Stable pairs, despite representing less than 2% of the total number of U2U pairs, generate a disproportionate amount of trading volume.

**Figure 3:**
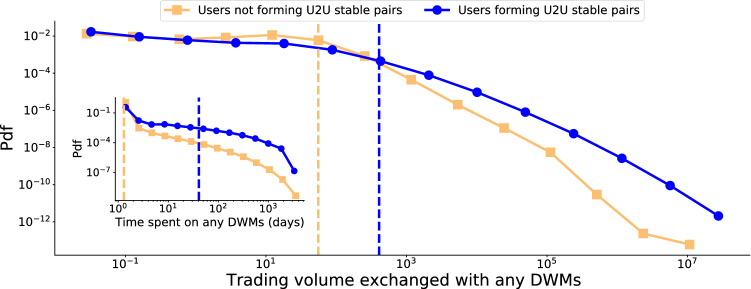
Role of users forming stable U2U pairs. (Main) PDFs of trading volume that users exchange with any DWMs. (Inset) PDFs of time spent by users on any DWMs. These distributions are explored for each of the 40 DWMs under consideration in Figure S5 and S6, respectively. Vertical lines represent median values of the respective distributions.

The high activity of users forming stable U2U pairs is not limited to the U2U network—these users are also the most active in trading with DWMs. Users in stable U2U pairs spend a median number of 41 days on DWMs versus a median of only one day for users without stable pairs. The two resulting distributions are significantly different (two-sided Kolmogorov-Smirnov test^35^: KS $$= 0.673$$, $$p<0.0001$$), see the inset of Fig. 3. When we look at the trading volume with DWMs, we find qualitatively similar results. Users in stable U2U pairs transact a median of $400 with DWMs, while other users transact only $56. The two resulting distributions are significantly different (KS $$= 0.438$$, $$p<0.0001$$), see Fig. 3. These results hold not only for full network but for every DWM in our data, see Figure S5 and S6.

### U2U network evolution

#### Formation of U2U stable pairs

Having mapped the behaviour of stable pairs, we now consider their temporal evolution. More specifically, we ask: How do stable pairs form? Do DWMs spur their creation? One possible hypothesis is that users meet for the first time while active on a DWM, i.e., after they have both traded with that DWM, see Table 1 and the nomenclature in Table 2. This can be considered as a plausible, and conservative, proxy for users who *met inside *a DWM (see Methods). A total of 37,129 users have met at least one other user inside a DWM. Their trading volume is about $417 million, and the percentage of users who met inside a DWM is proportional to the trading volume sent to DWMs (Spearman^36^: $$C=0.805$$, $$p<0.0001$$), see Fig. S7, meaning that larger DWMs are more likely to favour the encounter of users than smaller DWMs. Importantly, users who met inside a DWM transact more than those meeting outside a DWM. In particular, users who met inside a DWM trade a median of $2212 between themselves, almost twice the $1379 for users meeting outside the DWM (MNU $$= 1.863 \cdot 10^9$$, $$p<0.0001$$). Moreover, users who met inside a DWM tend to transact for significantly longer (median of 61 days) than users meeting outside (median of 50 days) (MNU $$= 2.099 \cdot 10^9$$, $$p<0.0001$$).

**Table 1:**
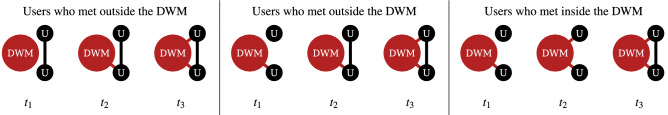
Formation mechanism of stable U2U pairs.

We compare the time at which the first transaction between a pair of users occurred with the time in which these users interacted with the same DWM. Each row in the figure indicates a possible temporal sequence, which we classify in two groups: users who *met outside* the DWM (first two columns) and users who *met inside* the DWM (last column).

#### Resilience of U2U stable pairs

**Figure 4:**
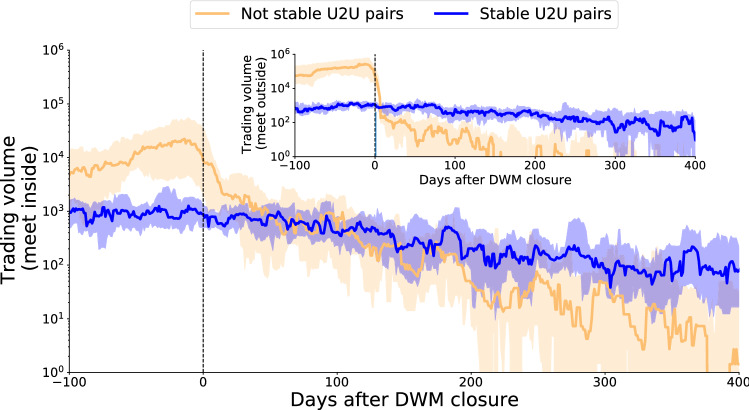
Resilience of stable U2U pairs after DWMs closure. Trading volume of U2U pairs surrounding active DWMs. (Main) U2U pairs meet who met inside aa DWM. (Inset) U2U pairs meet outside them. Plotted lines indicate the median value while bands represent the 95% confidence interval. Day zero corresponds to the day when the DWM closed. Negative and positive numbers indicate the days prior and after the closure, respectively. Only the 33 DWMs that closed during our time period are considered in the analysis.

Thus far, we have shown that users involved in stable trading relationships are also very active on DWMs, where they may meet new trading partners. But are DWMs and the U2U network truly interdependent? In particular, do stable pairs need the DWMs to survive? To answer these questions, we look at market closures, previously investigated to show how active users migrate to other existing DWMs^12^. Our dataset includes 33 closure events, which we study independently from one another by considering the evolution of the respective 33 marketplace ego networks. We find that non-stable U2U pairs sharply stop interacting immediately after the DWM closure, and therefore their existence is highly sensitive to the presence of the DWM. On the other hand, the trading volume of stable U2U pairs is only marginally affected by the disappearance of the DWM. As a result, while prior to DWM closures non-stable U2U pairs generate an overall trading volume that is 10 times higher than that of stable U2U pairs (since non-stable pairs are far more prevalent), within a few weeks after DWM closures the pattern is reversed: stable U2U pairs generate more trade volume than non-stable U2U pairs. Indeed, trading patterns of stable pairs are not significantly influenced by the sudden DWMs closure, and they very slowly decay over time, see Fig. 4.

**Figure 5:**
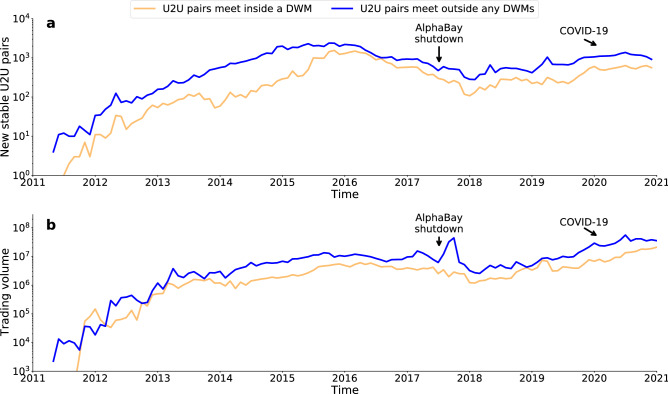
Temporal evolution of stable pairs. (**a**) Monthly number of new stable U2U pairs created. (**b**) Monthly trading volume of stable U2U pairs.

We have shown that the U2U network is resilient to abrupt external shocks, such as marketplace closures, and does not need the centralised structure of DWMs to survive. What about long-lasting systemic stress? To answer this question, we consider the impact that the COVID-19 pandemic has had on the evolution of stable U2U pairs. Previous studies reported that COVID-19 had a strong impact on DWMs due to delays and damage to the shipping infrastructure caused by border closures^37,38^. We start by investigating the number of new stable U2U pairs and their trading volume during the COVID-19 period. Users in stable pairs meeting both inside and outside DWMs have been growing over the last two years, since the shutdown of AlphaBay^9^, the largest DWM at the time. In 2020, a total of 6778 pairs of users in stable pairs met inside a DWM, corresponding to 192% of the 2019 level and to 255% of the 2018 level, see Fig. 5a. Pairs of users in stable pairs meeting inside a DWM traded for a total of $145 million in 2020, which corresponds to 252% of the 2019 level, and to 593% of the 2018 level, see Fig. 5b. We see similar trends for stable U2U pairs meeting outside DWMs. The impact of the COVID-19 pandemic has, however, had different phases, punctuated by the number and level of measures introduced around the world. For users in stable pairs who met both inside and outside DWMs, we find that during the first lockdowns in 2020 trading volume fell with respect to January of the same year, suggesting that they were negatively impacted by COVID-19 restrictions. After that, trading volume sharply increased over the whole of 2020, see Figure S8. The number of stable U2U pairs created each day was, however, steady over time during 2020, even though more U2U pairs were created compared to the same period in 2019, see Figure S9. Overall, stable U2U pairs have shown resilience to the systemic stress caused by COVID-19, suggesting, once again, that these trading relationships are fundamentally independent from the underlying DWMs.

## Discussion and conclusion

In this paper, we revealed the prevalence and structure of a large network of direct transactions between users who trade on the same DWM. We showed that some of the links of this user-to-user (U2U) network are ephemeral while other persist in time. We highlighted that a significant fraction of stable U2U pairs formed as their members were trading with the same DWM, suggesting that DWMs may play a role in promoting the formation of stable U2U pairs. We showed that the relationships between users forming stable pairs persist even after the DWM shuts down and are not significantly affected by COVID-19, suggesting overall resilience of stable pairs to external shocks.

Our study has several limitations. In particular, our dataset does not include any attributes related to either users or their Bitcoin transactions, such as, whether the transaction represents an actual purchase or not. Moreover, we do not have information about which users trade with other users on the same DWM. Finally, our coverage of DWMs, albeit extensive, may lack information on other trading forums where users could have met.

Our work has several policy implications. Our findings suggest that DWMs are much more than mere marketplaces^39^. DWMs are also communication platforms, where users can meet and chat with other users either directly—using Whatsapp, phone, or email—or through specialised forums. These direct interactions may favour the emergence of decentralised trade networks that bypass the intermediary role of the marketplace, similar to what is currently happening on Facebook, Telegram, and Reddit^16–20,23^, where users post products, negotiate item prices, and then trade directly. We estimate that the trading volume of U2U pairs meeting on DWMs is increasing, reaching a peak in 2020 (during the COVID-19 pandemic). By contrast, trading volume on DWMs was negatively affected by COVID-19, mainly due to shipping delays^37,38^. The reasons for the differential impact of COVID-19 on U2U trading vs. DWM trading are difficult to pin down. One hypothesis is that U2U pairs managed to find better shipping logistics; another hypothesis is that they were seen as a safer way to trade than DWMs at a time of crisis.

Our results also support recent recommendations of paying attention to individual sellers rather than entire DWMs^40^. Law enforcement agencies, however, have only recently started targeting individual sellers. The first operation took place in 2018 and successfully led to the arrest of 35 sellers^41^, while the largest operation to date occurred in 2020 and led to 179 arrests in six different countries^42^. Our study indicates that a much higher number of highly active DWM users, on the order of tens of thousands, is involved in transactions with other DWM users. Moreover, our analysis paves the way to a deeper understanding of U2U transactions in online marketplaces. Recent results have shown that transaction networks and activity on DWMs and regulated online marketplaces share several robust macroscopic properties^43^. One might therefore hypothesise that U2U trading is also a prevalent feature on regulated online marketplaces. While data on U2U transactions is far harder to obtain (as these transactions might involve a variety of commercial methods), there is clearly a need to better understand the dynamics and structure of trading relationships beyond what is observable on individual online marketplaces.

Overall, our study provides a first step towards the understanding of how users of DWMs collectively behave outside organised marketplaces. We believe that the results might suggest to researchers, practitioners, and law enforcement agencies that a shift in the attention from the evolution of DWMs to the behaviour of their users might facilitate the design of more appropriate strategies to counteract online trading of illicit goods.

## Data and methods

Additional considerations on our data and methods are available in Section S1.

### Data preprocessing

The raw dataset consists in transactions between Bitcoin addresses, which is initially preprocessed by Chainalysis Inc. (see Section S2). The resulting dataset consists in transactions between entities, that group together clusters of Bitcoin addresses. We consider only a subset of transactions in this dataset. Namely, we consider transactions made by the 40 entities representing the 40 DWMs under consideration, which directly interact with more than 16 million other entities, who are the users of these DWMs. Users interacting with other users form U2U pairs and we include them in our dataset. We discard single Bitcoin transactions below $0.01 or above $100,000, which are unlikely to show real purchases and minimise false positives. They may be attributed to a residual amount of Bitcoins in an address or transactions between two business partners where no good is actually given in return, respectively. The analysed dataset includes about 31 million transactions among more than 16 million users. Finally, we note that the same user can interact in multiple DWMs^12,13^. By definition, users that interact among themselves form U2U transactions. If the pair of users interact with multiple DWMs these U2U transactions are included in all related DWMs and counted multiple times. Therefore, the simple sum of all U2U transactions of each DWM is more than the sum of all unique U2U transactions. We count a total of 11 million transactions around all DWMs, that goes down to 9.9 million when multiple counting is avoided. Similarly, the simple sum of the single trading volumes surrounding all DWMs amounts to $33 billion, while the overall trading volume in all unique U2U pairs is $30 billion. Among the 40 large DWMs under consideration, 17 participate in at least one transaction in either 2020 or 2021, while the remaining 23 closed before 2020. Notably, our dataset includes Silk Road (the first modern DWM)^1^, Alphabay (once the leading DWM)^44^, and Hydra (currently the largest DWM in Russia)^12^. Other general statistics about our dataset can be found in the Section S3.

**Figure 6:**
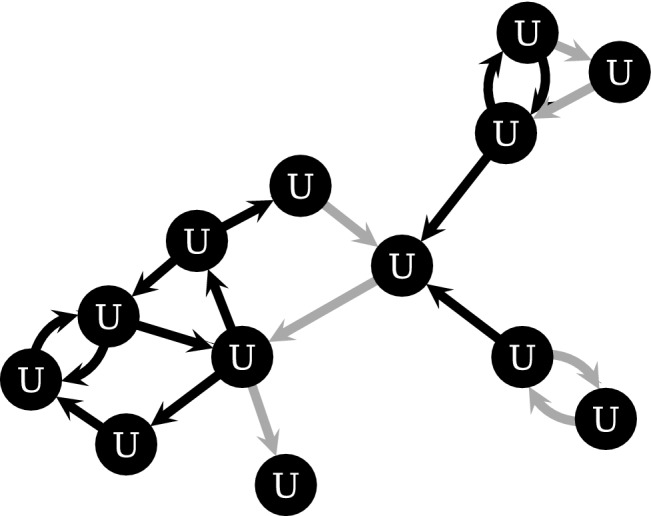
U2U network. The U2U network is formed by the entire set of interacting users (black and gray arrows with their respective users). Using the evolving activity-driven model^31^, U2U pairs are divided in either stable (black arrows and respective users) or non-stable (gray arrows and respective users).

### Detection of the U2U network

The detection of stable U2U pairs in the full network is done by using an evolving activity-driven model^31^, which introduced a statistically-principled methodology to detect the network backbone against what is expected from a proper null model. If a U2U pair occurs significantly more than what expected from the null model, it is labeled as stable, otherwise it is labelled as non-stable, see Fig. 6. The evolving activity-driven model is an appropriate methodology for large temporal networks^32^ and it is implemented in the Python 3 pip library TemporalBackbone^45^, where default parameter values have been used. As input parameter, we considered the full network, transactions from/to DWMs and U2U transactions between users (see Section S4).

### Users who met inside a DWM

We determine whether U2U pairs *meet* while active on a DWM by looking at the time occurrence of their first U2U transaction. This transaction can occur at three different moments in time: (i) At $t=t_1$, before both users interact with the same DWM (occurring at $t=t_2>t_1$ and $t=t_3>t_1$, respectively), as shown on the left hand side of Table 1. (ii) At $t=t_2$, when only one user has interacted with a specific DWM and the other user will do so at a later time, as shown in the middle column of Table 1. (iii) At $t=t_3$, when both users have interacted with the same DWM, as in the right column of Table 1. We classify these three chains of events in two groups. One group includes all pairs that *meet outside* any DWMs, which includes case (i) and case (ii), and the other group includes users that *meet inside* a DWM, described by case (iii). This last case constitutes a conservative proxy for users that meet inside a DWM. The proxy admits the possibility of false positives, since it counts users who met inside the same DWM without having interacted on it, as well as false negatives, since it does not take into account users who met inside a DWM without having ever interacted on it. The latter is arguably more significant, since it is possible that only one of the two users (the seller) has actually engaged in transactions with the DWM, while the other user, after seeing the seller’s profile on a DWM, has established a direct contact, through Whatsapp, email, or phone.

### Nomenclature of all groups considered

We provide the definition of all considered groups in Table 2.

**Table 2:** Nomenclature.

	Group	Description	Number of users
1.	Users who do not form stable U2U pairs	Users that either interact only with DWMs or form not stable U2U pairs	15,871,206
2.	Users who form stable U2U pairs	Users who form at least one stable U2U pair as detected by our chosen metholodogy^31^	106,648
2a.	Users who met outside DWMs	Users that form stable pairs and met at least one other user following the chain of events in Table 1 (first two columns)	88,828
2b.	Users who met inside a DWM	Users that form stable pairs and met at least one other user following the chain of events in Table 1 (last column)	37,129

Definitions of all groups the users are divided to based on their behaviour. Number of users in each group is given in the last column.

## Supplementary Information

Supplementary Information.

## Data Availability

All data needed to evaluate the conclusions in the paper are present in the paper. Additional data related to this paper may be requested from the authors.

## References

[1] Christin, N. Traveling the silk road: A measurement analysis of a large anonymous online marketplace. In *Proceedings of the 22nd International Conference on World Wide Web*, pp. 213–224 (2013).

[2] Dingledine, R., Mathewson, N. & Syverson, P. *Tor: The Second-Generation Onion Router*. Technical Report, Naval Research Lab Washington DC (2004).

[3] Nakamoto, S. *Bitcoin: A Peer-to-Peer Electronic Cash System*. Technical Report, Manubot (2008).

[4] Gwern. *Darknet Market Mortality Risks* (2019). https://www.gwern.net/DNM-survival. Accessed October 27th 2021.

[5] Broadhurst, R., Ball, M. & Jiang, C. Availability of COVID-19 related products on Tor darknet markets. *Aust. Inst. Criminol.* (2020).

[6] Bracci, A. *et al.* Dark web marketplaces and COVID-19: Before the vaccine. *EPJ Data Sci.* **10**, (2021). 10.1140/epjds/s13688-021-00259-w PMC7819623 33500876

[7] Bracci$$^+$$, A. *et al.* Dark web marketplaces and COVID-19: After the vaccines. arXiv preprint arXiv:2102.05470v4 (2021). (+) Contributed equally.

[8] Europol. *Operation Onymous* (2014).https://www.europol.europa.eu/activities-services/europol-in-action/operations/operation-onymous. Accessed October 27th 2021.

[9] Darknet takedown: Authorities shutter online criminal market AlphaBay (2017). https://www.fbi.gov/news/stories/alphabay-takedown. Accessed October 27th 2021. FBI.

[10] Isidore, C. *Feds Seize 1 Billion in Bitcoins they say were Stolen from Silk Road* (2020). https://edition.cnn.com/2020/11/06/business/bitcoin-seized-silk-road-ulbricht/index.html. Accessed January 4th 2021. CNN.

[11] Chainalysis. *The Chainalysis 2021 Crypto Crime Report* (2021). https://go.chainalysis.com/2021-Crypto-Crime-Report.html. Accessed October 27th 2021.

[12] ElBahrawy A (2020). Collective dynamics of dark web marketplaces. Sci. Rep..

[13] Hiramoto N, Tsuchiya Y (2020). Measuring dark web marketplaces via bitcoin transactions: From birth to independence. For. Sci. Int. Digit. Investig..

[14] Buxton J, Bingham T (2015). The rise and challenge of dark net drug markets. Policy Brief.

[15] Maddox A, Barratt MJ, Allen M, Lenton S (2016). Constructive activism in the dark web: Cryptomarkets and illicit drugs in the digital ‘demimonde’. Inf. Commun. Soc..

[16] Oksanen, A. *et al.* Illicit drug purchases via social media among American young people. In *International Conference on Human–Computer Interaction*, pp. 278–288 (Springer, 2020).

[17] German police seized nine telegram-based drug markets (2020). https://darknetlive.com/post/german-police-seized-nine-telegram-based-drug-markets/. Accessed October 27th 2021. DarknetLive.

[18] Sung Y-H, Lee W-H, Leung FK-W, Fong JJ (2021). Prevalence of illegal turtle trade on social media and implications for wildlife trade monitoring. Biol. Conserv..

[19] Childs, A., Bull, M. & Coomber, R. Beyond the dark web: Navigating the risks of cannabis supply over the surface web. *Drugs Educ. Prevent. Policy* 1–12 (2021).

[20] Kwon KH, Shao C (2021). Dark knowledge and platform governance: A case of an illicit e-commerce community in reddit. Am. Behav. Sci..

[21] Barratt, M. J., Lenton, S., Maddox, A. & Allen, M. “what if you live on top of a bakery and you like cakes?” –drug use and harm trajectories before, during and after the emergence of silk road. *Int. J. Drug Policy* **35**, 50–57 (2016). 10.1016/j.drugpo.2016.04.006 27157539

[22] Munksgaard R, Martin J (2020). How and why vendors sell on cryptomarkets. Trends Issues Crime Crim. Justice.

[23] Bakken SA, Demant JJ (2019). Sellers-risk perceptions in public and private social media drug markets. Int. J. Drug Policy.

[24] for Drugs, E. M. C., Addiction, D., & Europol. Drugs and the darknet: Perspectives for enforcement, research and policy (2017).

[25] Organization, W. H. World drug report 2019. *United Nations publication. Sales No. E* **19**, (2019).

[26] Gwern. *Updated: list of dark net markets (Tor & I2P)* (2020). https://www.gwern.net/DNM-survival. Accessed October 27th 2021.

[27] Lee, S. *et al.* Internet Society Cybercriminal minds: An investigative study of cryptocurrency abuses in the dark web. In *Network and Distributed System Security Symposium*, 1–15 (Internet Society, 2019).

[28] Foley S, Karlsen JR, Putniņš TJ (2019). Sex, drugs, and Bitcoin: How much illegal activity is financed through cryptocurrencies?. Rev. Financ. Stud..

[29] Baur, A. W., Bühler, J., Bick, M. & Bonorden, C. S. Cryptocurrencies as a disruption? Empirical findings on user adoption and future potential of Bitcoin and co. In *Conference on e-Business, e-Services and e-Society*, 63–80 (Springer, 2015).

[30] Saiedi, E., Broström, A. & Ruiz, F. Global drivers of cryptocurrency infrastructure adoption. *Small Bus. Econ.* **1–54**, (2020).

[31] Nadini M, Bongiorno C, Rizzo A, Porfiri M (2020). Detecting network backbones against time variations in node properties. Nonlinear Dyn..

[32] Nadini M, Rizzo A, Porfiri M (2020). Reconstructing irreducible links in temporal networks: Which tool to choose depends on the network size. J. Phys. Complex..

[33] Wilcoxon, F. Individual comparisons by ranking methods. In *Breakthroughs in Statistics*, 196–202 (Springer, 1992).

[34] Mann, H. B. & Whitney, D. R. On a test of whether one of two random variables is stochastically larger than the other. *Ann. Math. Stat.* **50–60**, (1947).

[35] Massey FJ (1951). The Kolmogorov–Smirnov test for goodness of fit. J. Am. Stat. Assoc..

[36] Spearman, C. The proof and measurement of association between two things. *Appleton-Century-Crofts* (1961).

[37] Bergeron A, Décary-Hétu D, Giommoni L (2020). Preliminary findings of the impact of COVID-19 on drugs crypto markets. Int. J. Drug Policy.

[38] *Covid is Causing Shipping Issues, But Natural Competitive Forces are Causing Darknet Market Consolidation* (2020). https://blog.chainalysis.com/reports/darknet-markets-cryptocurrency-2020. Accessed October 27t 2021. Chainalysis Team.

[39] Gupta, A., Maynard, S. B. & Ahmad, A. The dark web phenomenon: A review and research agenda. arXiv preprint arXiv: 2104.07138 (2021).

[40] Horton-Eddison, M., Shortis, P., Aldridge, J. & Caudevilla, F. Drug cryptomarkets in the 2020s: Policy, enforcement, harm, and resilience. Global Drug Policy. *Observatory* (2021).

[41] of Public Affairs, O., First nationwide undercover operation targeting darknet vendors results in arrests of more than 35 individuals selling illicit goods and the seizure of weapons, drugs and more than \$23.6 million (2018). https://www.justice.gov/opa/pr/first-nationwide-undercover-operation-targeting-darknet-vendors-results-arrests-more-35. Accessed October 27th 2021. Department of Justice, United States.

[42] Team, E. *International Sting Against Dark Web Vendors Leads to 179 Arrests* (2020). https://www.europol.europa.eu/newsroom/news/international-sting-against-dark-web-vendors-leads-to-179-arrests. Accessed October 27th 2021. Europol.

[43] Bracci, A. *et al.* Macroscopic properties of buyer–seller networks in online marketplaces. arXiv preprint arXiv: 2112.09065 (2021). 10.1093/pnasnexus/pgac201 PMC9802486 36714880

[44] Van Buskirk, J., Naicker, S., Bruno, R., Breen, C. & Roxburgh, A. Drugs and the internet. *The National Illicit Drug Indicators Project* (2016).

[45] Nadini, M. *Temporalbackbone* ( 2021). https://pypi.org/project/TemporalBackbone/. Accessed October 27th 2021. Python pip 3 library: A tool to detect the backbone in temporal networks.

