# Supplementary material for: Emergence and structure of decentralised trade networks around dark web marketplaces
Source: Sci Rep. 2022 Mar 31;12:5425. doi: 10.1038/s41598-022-07492-x (PMC8971429; doi:10.1038/s41598-022-07492-x)
Supplement: Supplementary file 1 — Supplementary Information. [file 41598_2022_7492_MOESM1_ESM.pdf]

Emergence and structure of decentralised trade networks around dark web marketplaces

Matthieu Nadini[1,2], Alberto Bracci[1], Abeer ElBahrawy[3], Philip Gradwell[3], Alexander Teytelboym[4], and Andrea Baronchelli[1,2,5,*]

[1]Department of Mathematics, City University of London, EC1V 0HB, London, UK
[2]The Alan Turing Institute, British Library, 96 Euston Road, NW12DB, London, UK
[3]Chainalysis Inc, NY, USA
[4]Department of Economics and INET Oxford, University of Oxford, Oxford OX1 3UQ, UK
[5]UCL Centre for Blockchain Technologies, University College London, London, UK
[*]abaronchelli@turing.ac.uk

ABSTRACT

In this Supplementary Information, we show additional information on our methods as well as additional analyses that support our findings.

S1 Additional data and methods

Identification of real identities performing Bitcoin transactions. The trading volume of DWMs has been steadily increasing and exceeded \$1.5 billion for the first time in 2020[1]. The vast majority of such trading has occurred in Bitcoin, which is the most popular cryptocurrency to date. Its worldwide adoption has further increased in 2021, jumping over 880% with respect to 2020[2]. Bitcoin allows users to use pseudonym (public address) instead of their real identities. Users can create a new pseudonym at each transaction, requiring only a computer and an internet connection. However, various heuristics exist to cluster addresses together to recover the real identity behind pseudonyms[3]. In our dataset, this process is done by Chainalysis Inc. (see Section S2). In the dataset, real entities represent DWMs, users of DWMs, or other entities interacting with these users. Transactions to and from Bitcoin trading exchanges are removed, because our primary interest entails the study of direct interactions between DWMs and single users. The dataset comprises of 40 DWMs, for a total of 149 transactions among 57 million real entities. Each Bitcoin transaction has an associated timestamp t, indicating the time at which the transaction occurred. The dataset is sparse, with 54.6% of all entities performing a transaction only. The conversion from Bitcoin to dollars is done using the price of Bitcoin at the time of the transaction.

Evaluation of coefficients of the trend line in Figure 2(a). The coefficients $a = 1.06$ and $b = 0.70$ of the trend line $y = x^a 10^{-b}$ in Figure 2(a) are in good agreement with the empirical data, $R^2 = 0.969$, and evaluated as follows. First, the equation is transformed to $Y = aX - b$, where $Y = \log_{10} y$ and $X = \log_{10} x$. The linear equation fitted against real data and coefficients a and b computed by minimizing the sum of squares.

Statistical analysis. We compare the median of two paired distributions using the two-sided Wilcoxon test[4]. It is a non-parametric statistical test and verifies the null hypothesis that two paired samples come from distributions with the same median. If distributions are not paired, we use the Mann-Whitney-U test to assess statistical differences of the medians of two distributions[5]. We compare two distributions using the Kolmogorov-Smirnov test[6] on two samples. It tests the null hypothesis that 2 independent samples are drawn from the same continuous distribution. We evaluate the correlation between two sets of values using the Spearman rank-order correlation coefficient[7]. It is a correlation coefficient that does not assume normally distributed values and varies between -1 and 1: with -1 implying a negative correlation, 0 no correlation, and 1 a positive correlation.

S2 Dark web marketplaces and identification of real identities performing Bitcoin transactions.

S2.1 Dark web marketplaces

DWMs are in many ways similar to other online marketplaces. They have strict policies that every user must follow. For instance, in some DWMs are banned categories of products, like human trafficking, contract killing, weapons, or COVID-19 fake vaccines[8,9]. Registration is required for all sellers, and sometimes also for buyers. Certified sellers can advertise their products. They have a reputation, which is based on buyers' reviews[10,11]. They are also responsible for delivering the products, sometimes with a tracking number attached, and may offer refunds or reshipment. Buyers are free to look at the listings and sometimes can ask questions directly to the relative seller[12,13]. Payments are often protected by escrow services. These are third-party services, which guarantee that buyers can safely have their money refunded. Users' on DWMs constitute an active community. Numerous are websites and forums where users can share their experience and get advice on the most trustworthy DWMs and sellers, such as Dread[14], Raptor.life[15], DarkNetLive[16], and DarkFail[17].

DWMs have some unique features as well. They sell several kinds of illicit products, like drugs, fake IDs, and medicines[18–20]. They are not accessible by standard web search-engines, but operate online in an encrypted part of the Internet[21]. Potential buyers can easily access to DWMs using specialized browsers, like The Onion Router (Tor)[22], and anonymously trade illicit goods using cryptocurrencies, like Bitcoin[23]. Bitcoin is currently the most popular cryptocurrency on DWMs[24–26] and its adoption is growing in the regular economy as well. Its infrastructure seems to ensure complete anonymity to its users. If a proper technique is adopted, however, there are chances to link the Bitcoin blockchain (that is, the entire Bitcoin transaction history) with the user's real identity[3]. When the Bitcoin blockchain is successfully linked to a real identity, the records of past, present, and future Bitcoin transactions is traceable, easily accessible, and can be used by companies, law enforcement agencies, and researchers.

S2.2 Identification of real identities performing Bitcoin transactions

The raw, anonymized Bitcoin blockchain can be publicly accessed through Bitcoin core[27] or third-party APIs such as Blockchain.com[28]. It contains information about origin and destination addresses, as well as time and amount of the transactions. In order to contrast traceability of the real identity, an user is likely to use multiple addresses. A new address is often generated in each transaction. Grouping the addresses in clusters reduces the complexity of the Bitcoin blockchain and challenge users' anonymity[29]. Given that millions of Bitcoin addresses are currently active and many others are continuously being generated, a clustering approach primarily based on manual annotation is not feasible. Various heuristics, instead, have been proposed[29–32]. They were successful in grouping Bitcoin addresses and associate them to cluster of real entities. For instance, in[29], the authors were able to find a connection between a set of large transactions and a single one, which was dated in November 2010. In[30], the authors applied to a daily university setting the privacy protocol recommended in Bitcoin transactions, finding that almost 40% of the real identities would be recovered. Another work showed the presence of "super clusters" of entities, which marked macro-variations in the evolution of the Bitcoin economy[31]. The primary reasons behind the effectiveness of heuristic clustering are: "address reuse, avoidable merging, super-clusters with high centrality, and the incremental growth of address clusters"[32].

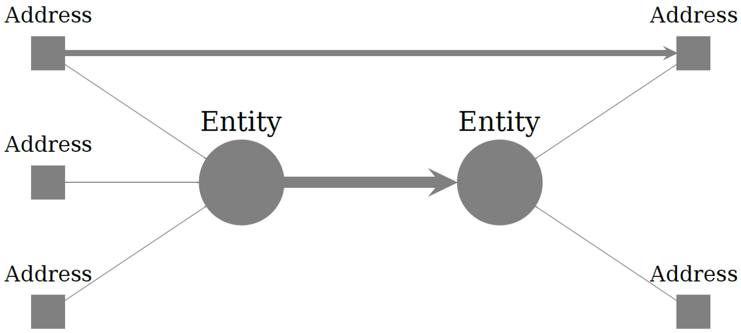

Figure S1. Identification of real entities in the Blockchain. End goal of Bitcoin transactions clustering techniques: mapping a series of Bitcoin addresses to real entities. In this example, an address sends Bitcoins to another address. Thanks to the identification process, the two addresses are associated with two real entities. The Bitcoin transaction between the two entities becomes traceable and transparent.

The end goal of clustering Bitcoin addresses is to map them to single, real entities, as shown in Figure S1. To achieve this goal, however, heuristic clustering techniques should be improved. Manual annotation has shown a valuable potential[33]. It consists on gathering publicly available Bitcoin addresses, like the Wikimedia Foundation one[34], and engage through direct interaction with unknown Bitcoin addresses. If some real entities are known, it is easier to associate the remaining Bitcoin addresses to other real identities. In the last few years, companies specialising in Bitcoin analytics have started to leverage previous methodologies[29–33] to unveil real entities. The leading company in analysing Bitcoin transactions on DWMs is Chainalysis Inc.[35], which has also aided several federal investigations. For instance, it supported the United States Internal Revenue Service (IRS) in tracking Bitcoin transactions[36] and the FBI in the Twitter hack[37]. Chainalsysis clusters Bitcoin transactions in groups by combining previous methodologies[29–32] and real entities are unveiled with an approach similar to[33]. In the dataset, real entities represent DWMs, users of DWMs, or other real entities interacting with these users. Chainalysis aims at minimizing the false positives, who may lead to wrongly associate a real entity with illicit activities. If a Bitcoin address cannot be uniquely ascribed to a real entity, it is included in our dataset as an independent and unnamed entity. Only a fraction of the entities in our dataset thus represent named and real entities, which identity is known. Given that there are millions of entities in our dataset, it is impossible to identify all the corresponding real identities. After the identification process is completed, to each real entity is associated a string of numbers and the dataset re-anonymized. Transactions to and from Bitcoin trading exchanges are also removed, because our primary interest entails the study of direct interactions between real entities. As a result, the dataset analysed in this article is created by using state-of-the-art clustering techniques to identify addresses owned by the same user and label real entities.

S3 General statistics of the 40 DWMs under consideration

Name	Transactions with a DWM		U2U transactions	
	Users (sent; received; total)	Trading volume in millions (sent; received; total)	Users (sent; received; total)	Trading volume in millions (total)
Abraxas	(95,642; 21,500; 111,003)	(21.85; 27.23; 49.09)	(28,588; 25,546; 44,151)	61.92
Agora	(462,106; 119,221; 537,983)	(141.3; 132.8; 274.1)	(168,248; 151,699; 252,984)	558.0
AlphaBay	(1,658,059; 334,154; 1,898,850)	(537.1; 568.6; 1,106)	(524,783; 422,881; 776,183)	1581
Apollon	(68,373; 13,954; 79,307)	(12.90; 16.59; 29.50)	(19,468; 17,290; 29,900)	49.38
Basetools	(119,114; 347; 119,461)	(4.712; 6.727; 11.44)	(32,191; 34,169; 50,939)	63.23
Benumb Shop	(27,229; 343; 27,556)	(3.929; 5.027; 8.956)	(5,499; 5,654; 8,985)	21.73
BitBazaar	(20,805; 150; 20,931)	(2.681; 4.425; 7.106)	(6,939; 6,569; 10,126)	14.13
Black Bank	(52,783; 15,147; 64,131)	(11.41; 11.78; 23.19)	(15,843; 13,486; 24,291)	31.11
Blue Sky	(16,002; 10,140; 22,616)	(3.225; 3.786; 7.011)	(9,763; 6,149; 12,108)	10.86
Buybest	(334,741; 3,004; 337,556)	(24.45; 7.490; 31.94)	(57,001; 59,131; 99,390)	132.4
Bypass Shop	(861,716; 8,118; 869,593)	(65.66; 54.36; 120.0)	(176,905; 174,151; 288,745)	804.3
DarkMarket	(176,141; 13,554; 183,010)	(27.25; 36.62; 63.87)	(72,923; 67,621; 105,416)	166.7
Dream	(466,511; 45,399; 507,837)	(57.64; 72.95; 130.6)	(109,871; 70,706; 154,873)	287.3
Empire	(405,202; 9,690; 413,858)	(64.38; 56.84; 121.2)	(63,431; 56,415; 103,886)	287.4
Evolution	(216,604; 34,512; 240,713)	(47.58; 50.15; 97.73)	(77,331; 72,711; 115,496)	236.7
FEshop	(1,134,456; 5,858; 1,140,275)	(64.67; 48.83; 113.5)	(244,318; 261,489; 420,040)	834.4
Flugsvamp 2.0	(104,385; 21,201; 119,893)	(23.01; 38.20; 61.22)	(29,215; 23,079; 41,047)	144.2
Flugsvamp 3.0	(217,083; 20,773; 234,563)	(39.34; 52.78; 92.12)	(52,075; 49,881; 81,527)	473.8
FuLLzShOp	(21,716; 9; 21,726)	(3.937; 4.510; 8.447)	(4,147; 4,496; 7,209)	10.07
Hansa	(330,565; 73,202; 358,120)	(60.64; 55.91; 116.6)	(153,567; 127,514; 209,717)	76.60
Hydra	(4,031,013; 666,075; 4,584,339)	(1,868; 1,810; 3,678)	(2,447,548; 2,099,320; 3,124,366)	20,840
Joker's Stash	(806,089; 1,090; 807,140)	(153.0; 49.95; 203.0)	(154,872; 156,689; 260,832)	926.2
LuxSocks.ru	(326,159; 186; 326,340)	(8.123; 5.573; 13.70)	(59,638; 66,011; 97,705)	175.8
Matanga	(57,354; 633; 57,963)	(5.882; 7.775; 13.66)	(10,637; 10,328; 17,632)	96.35
Middle Earth	(38,017; 9,206; 45,629)	(8.361; 9.151; 17.51)	(8,091; 7,603; 12,990)	18.68
MrGreen.ws	(44,918; 176; 45,094)	(8.244; 6.176; 14.42)	(6,298; 5,912; 10,501)	14.44
Nightmare	(37,844; 3,524; 40,894)	(5.697; 7.371; 13.07)	(8,830; 6,277; 12,905)	25.83

Table S1. General statistics of DWMs, part 1. Some DWMs are presented here, the others are available in Table S2. The terms "sent" and "received" always refer to transactions made by users. The trading volume indicates millions of dollars. The amount of dollars sent and received by users through U2U transactions is equivalent to the total.

Name	Interactions with DWM		U2U interactions	
	Users (sent; received; total)	Trading volume in millions (sent; received; total)	Users (sent; received; total)	Trading volume in millions (total)
Nucleus	(205,043; 53,571; 247,884)	(56.59; 61.68; 118.3)	(62,577; 52,829; 93,279)	156.9
Pandora	(35,667; 8,723; 41,718)	(8.401; 8.561; 16.96)	(11,119; 8,964; 15,944)	26.37
Russian Anonymous	(740,625; 36,161; 769,228)	(80.24; 95.94; 176.2)	(363,773; 331,811; 493,766)	1866
San-Wells	(51,795; 2,858; 54,633)	(6.335; 5.755; 12.09)	(8,227; 7,841; 13,679)	15.36
Sheep	(38,068; 7,634; 42,673)	(10.81; 11.47; 22.29)	(12,007; 10,288; 182,90)	49.87
Silk Road	(382,534; 72,344; 429,284)	(130.2; 149.7; 279.9)	(163,376; 157,113; 243,441)	671.4
Silk Road 2	(222,666; 47,528; 254,830)	(66.83; 71.92; 138.7)	(73,116; 66,019; 111,387)	259.8
Silk Road 3.1	(59,894; 15,413; 70,078)	(9.054; 13.49; 22.54)	(22,160; 18,570; 32,491)	21.80
TradeRoute	(103,517; 14,080; 112,634)	(16.97; 17.04; 34.01)	(27,901; 22,287; 41,869)	67.72
Unicc	(2,004,236; 559; 2,004,789)	(147.8; 84.61; 232.4)	(473,969; 490,794; 780,282)	1,673
Valhalla	(82,507; 8,218; 89,214)	(8.933; 9.811; 18.74)	(25,755; 32,687; 45,297)	51.49
Wall Street	(334,871; 25,352; 347,842)	(48.15; 53.16; 101.3)	(148,262; 127,370; 203,176)	163.5
xDedic	(27,956; 885; 28,736)	(3.552; 3.838; 7.389)	(4,785; 4,767; 7,685)	12.70

Table S2. General statistics of DWMs, part 2. Some DWMs are presented here, the others are available in Table S1. The terms "sent" and "received" always refer to transactions made by users. The trading volume indicates millions of dollars. The amount of dollars sent and received by users through U2U transactions is equivalent to the total.

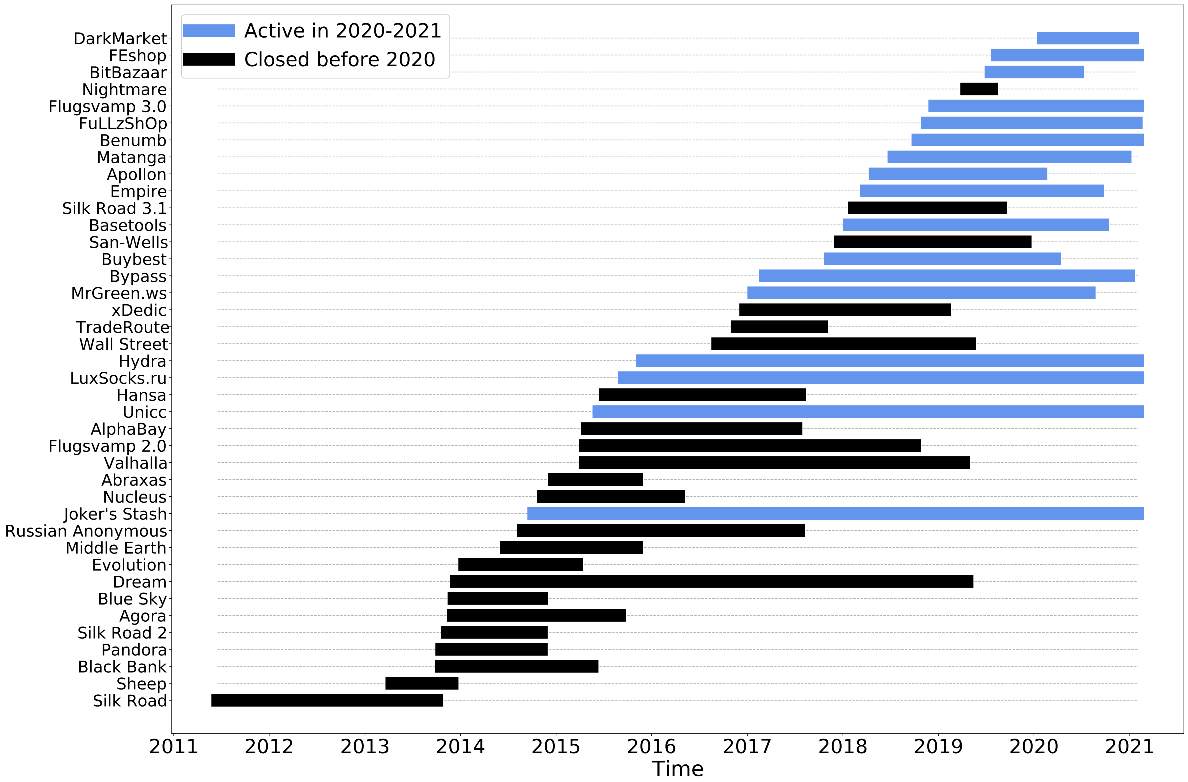

Figure S2. Lifetime of DWMs in our dataset. Time interval between the first and last transaction of each DWM. A total of 17 DWMs participated in at least one transactions in either 2020 or 2021, while 23 closed before 2020.

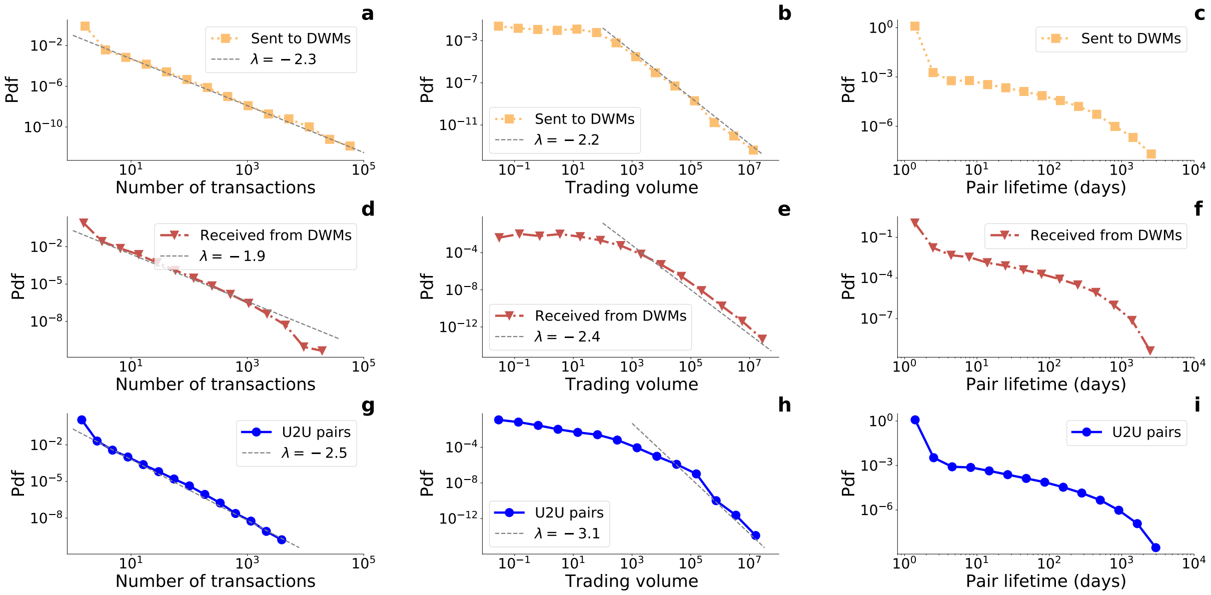

Figure S3. Key distributions of the full network. Probability distribution function (pdf) about the number of transactions of each pair of entities (a)-(d)-(g), their trading volume (b)-(g)-(h), and their lifetime computed as time difference between their last and first transaction (c)-(f)-(i).

S4 Detection of stable pairs in temporal and directed networks

Here, we summarize the methodology of detecting the backbone of stable pairs in temporal and undirected networks as introduced in[38], and show how it can be easily adapted to tackle the analysis of directed temporal networks. The methodology follow three sequential steps: (i) determine the interval partition, (ii) estimate models' parameters, over successive intervals, and (iii) run a statistical filter, which removes all pairs explained by the null hypothesis and retain stable pairs. The analysed temporal network, either directed or undirected, of N nodes evolves in an observation window composed of $T \gg 1$ time steps, labeled as $t = 1,...,T$. At each time step t, entities interact among themselves and form a time-varying network of interactions, described by a binary adjacency matrix that varies in time $A(t)$.

S4.1 Temporal and undirected networks

Interval partition. The overall observation window is divided in successive and disjoint intervals using an auxiliary method, namely, the Bayesian Block method[39]. It takes as input the total number of temporal pairs created in the entire network at time t

$$\Omega^{\text{ts}}(t) = \sum_{i,j=1;i\leq j}^{N} A_{ij}^{\text{ts}}(t), \tag{1}$$

where the superscript "ts" indicates that these variables are estimated from the time series and $A_{ij}^{\text{ts}}(t)$ is the ijth entry of the estimated adjacency matrix at time t. The Bayesian Block method returns the interval partition, which divides the overall time window T into I disjoint intervals indexed by $\Delta = 1,\ldots,I$, that contain a uniform total number of connections. From the knowledge of the interval partition, the length, $\tau(\Delta)$, of the generic Δth interval is obtained with the following closure relation: $\sum_{\Delta=1}^{I} \tau(\Delta) = T$.

Parameter estimation. According to the null hypothesis, pair of entities i and j are expected to interact proportional to the their individual activities at time t. That is, the probability that entities i and j interact at time t is a binomial random variable defined as

$$p_{ij}(t) \equiv a_i(t)a_j(t), \tag{2}$$

where $a_i(t)$ and $a_j(t)$ are piece-wise constant activities, which represent the propensity of creating interactions at time t. The estimation of piece-wise constant activities is carried out analysing each of the I intervals separately. The activity of entity i at time $t \in \left[t_{\text{in}(\Delta)}, t_{\text{in}(\Delta)} + \tau(\Delta) - 1\right]$ is computed through the following frequency count:

$$a_i(t) = \frac{s_i^{\text{ts}}(\Delta)}{\sqrt{2W^{\text{ts}}(\Delta)\tau(\Delta)}}, \tag{3}$$

where $s_i^{\text{ts}}(\Delta)$ and $W^{\text{ts}}(\Delta) \gg 1$ are the total number of pairs generated by entity i in the Δth and the total number of temporal pairs generated in the network in the Δth interval, respectively. These variables are computed from the adjacency matrix $A^{\text{ts}}(t)$, as, $s_i^{\text{ts}}(\Delta) = \sum_{j=1}^{N} \sum_{t=t_{\text{in}(\Delta)}}^{t_{\text{in}}(\Delta)+\tau(\Delta)-1} A_{ij}^{\text{ts}}(t)$, and $W^{\text{ts}}(\Delta) = \frac{1}{2}\sum_{i=1}^{N} s_i^{\text{ts}}(\Delta)$. Once the activities are estimated according with Eq. (3), the probability in Eq. (2) can be calculated.

Statistical filter. The statistical filter compares expected number of connections between entity i and entity j, $\mathrm{E}\left[\overline{w}_{ij}\right]$, with observations from the time series, $\overline{w}_{ij}^{\text{ts}} = \sum_{t=1}^{T} A_{ij}^{\text{ts}}(t)$. The expected number of connections between entities i and j in the overall time window T is determined by the sum of the binomial random variables given in Eq. (2)

$$\mathrm{E}\left[\overline{w}_{ij}\right] = \sum_{t=1}^{T} p_{ij}(t) = \sum_{\Delta=1}^{I} \frac{s_i^{\text{ts}}(\Delta)s_j^{\text{ts}}(\Delta)}{2W^{\text{ts}}(\Delta)}, \tag{4}$$

where we have used the estimation of activity in Eq. (3) and summed over all intervals. Although the sum of non-identical binomial random variables in Eq. (4) is a Poisson binomial distribution, the Poisson distribution is an

appropriate approximation for long time series. The probability that the observed weight, $\overline{w}_{ij}^{\mathrm{ts}}$, could be explained by the relative expected weight, $\mathrm{E}\left[\overline{w}_{ij}\right]$ in Eq. (4), is computed according to the cumulative function of the Poisson distribution

$$\alpha_{ij} \equiv 1 - \sum_{x=0}^{\overline{w}_{ij}^{\mathrm{ts}}-1} P\left(x; \mathrm{E}\left[\overline{w}_{ij}\right]\right), \tag{5}$$

where $P\left(x; \mathrm{E}\left[\overline{w}_{ij}\right]\right)$ indicates the Poisson distribution with random variable x and expected value $\mathrm{E}\left[\overline{w}_{ij}\right]$. Equation (5) represents the p-value α_{ij}: when the p-value is below a pre-defined threshold, the pair ij is significant and included in the backbone network. The same statistical test is repeated for all pairs of entities ij observed at least once in the overall temporal evolution.

S4.2 Temporal and directed networks

With little modifications, the above methodology can be used to filter temporal and directed networks.

Interval partition. The interval partition is obtained by using the Bayesian Block method as above. The total number of temporal pairs created in the entire network at time t is

$$\Omega^{\mathrm{ts}}(t) = \sum_{i,j=1}^{N} A_{ij}^{\mathrm{ts}}(t), \tag{6}$$

where not pairs are directed, while in Eq. (1) undirected, thereby explaining the different ranges in the summations.

Parameter estimation. In directed networks, the probability that entity i contacts at random entity j at time t is defined as

$$p_{i\to j}(t) \equiv a_i(t) b_j(t). \tag{7}$$

where $a_i(t)$ is the activity of entity i at time t and $b_j(t)$ the attractiveness of entity j at time t. The activity was already defined in Eq. (2), while the attractiveness represent the propensity of receiving connections at time t. If $a_i(t) = b_i(t)\ \forall i,t$ (for all entities in the network and at all time), Eq. (7) becomes equivalent to Eq. (2). However, care should be placed in their interpretation, whereby Eq. (7) generates a directed pair from entity i to entity j, while Eq. (2) can only lead to an undirected pair.

In the generic Δth interval, defining the time window $t \in \left[t_{\mathrm{in}(\Delta)}, t_{\mathrm{in}(\Delta)} + \tau(\Delta) - 1\right]$, piece-wise constant activities and attractivenesses are estimated directly from the time series, similarly to what done in the undirected case in Eq. (3)

$$a_i(t) = \frac{s_{\mathrm{out},i}^{\mathrm{ts}}(\Delta)}{\sqrt{W^{\mathrm{ts}}(\Delta)\tau(\Delta)}} \qquad b_i(t) \quad = \frac{s_{\mathrm{in},i}^{\mathrm{ts}}(\Delta)}{\sqrt{W^{\mathrm{ts}}(\Delta)\tau(\Delta)}}, \tag{8}$$

where $s_{\mathrm{out},i}^{\mathrm{ts}}(\Delta)$, $s_{\mathrm{in},i}^{\mathrm{ts}}(\Delta)$, and $W^{\mathrm{ts}}(\Delta) \gg 1$, are the total incoming strength of entity i in the Δth interval, outgoing strength of entity i in the Δth interval, and the total number of directed, temporal pairs generated in the network in the Δth interval, respectively. These variables are computed from the adjacency matrix $A^{\mathrm{ts}}(t)$, that is, $s_{\mathrm{out},i}^{\mathrm{ts}}(\Delta) = \sum_{j=1}^{N} \sum_{t=t_{\mathrm{in}(\Delta)}}^{t_{\mathrm{in}}(\Delta)+\tau(\Delta)-1} A_{ij}^{\mathrm{ts}}(t)$, $s_{\mathrm{in},i}^{\mathrm{ts}}(\Delta) = \sum_{i=1}^{N} \sum_{t=t_{\mathrm{in}(\Delta)}}^{t_{\mathrm{in}}(\Delta)+\tau(\Delta)-1} A_{ij}^{\mathrm{ts}}(t)$, and $W^{\mathrm{ts}}(\Delta) = \sum_{i=1}^{N} s_{\mathrm{out},i}^{\mathrm{ts}}(\Delta)$. Once the activity and attractiveness are estimated according with Eq. (8), the probability in Eq. (7) can be evaluated.

Statistical filter. Similar to Eq. (4), the expected number of pairs from entity i to entity j is computed by summing the probability in Eq. (7) for all time instants t

$$\mathrm{E}\left[\overline{w}_{i\to j}\right] = \sum_{t=1}^{T} p_{i\to j}(t) = \sum_{\Delta=1}^{I} \frac{s_{\mathrm{out},i}^{\mathrm{ts}}(\Delta) s_{\mathrm{in},j}^{\mathrm{ts}}(\Delta)}{W^{\mathrm{ts}}(\Delta)}. \tag{9}$$

The probability that the observed weight, $\overline{w}^{\mathrm{ts}}_{i\to j}$, is explained by the expected weight, $\mathrm{E}\left[\overline{w}_{i\to j}\right]$ in Eq. (9), is computed according to the cumulative function of the Poisson distribution

$$\alpha_{i\to j} \equiv 1 - \sum_{x=0}^{\overline{w}^{\mathrm{ts}}_{i\to j}-1} P\left(x;\mathrm{E}\left[\overline{w}_{i\to j}\right]\right). \tag{10}$$

Equation (10) represents the p-value $\alpha_{i\to j}$, which is used to assess whether the directed pair $i \to j$ is significant. The same statistical test has to be repeated for directed pairs observed at least once in the overall temporal evolution. For undirected networks, Eq. (10) is equivalent to Eq. (5).

S5 Additional simulations

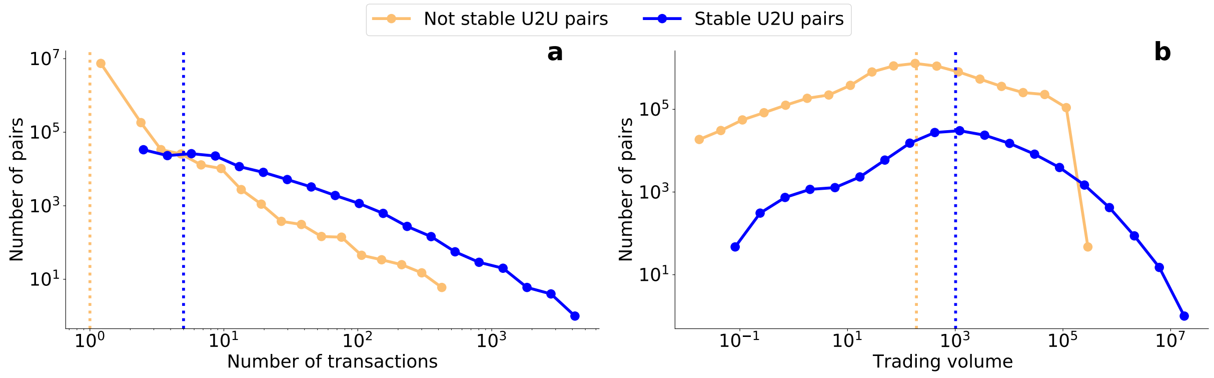

Figure S4. Statistics of U2U pairs. (a) Number of stable and non-stable U2U pairs with a given number of transactions. (b) Number of stable and non-stable U2U pairs with a given trading volume. Vertical lines represent median values of the respective distributions.

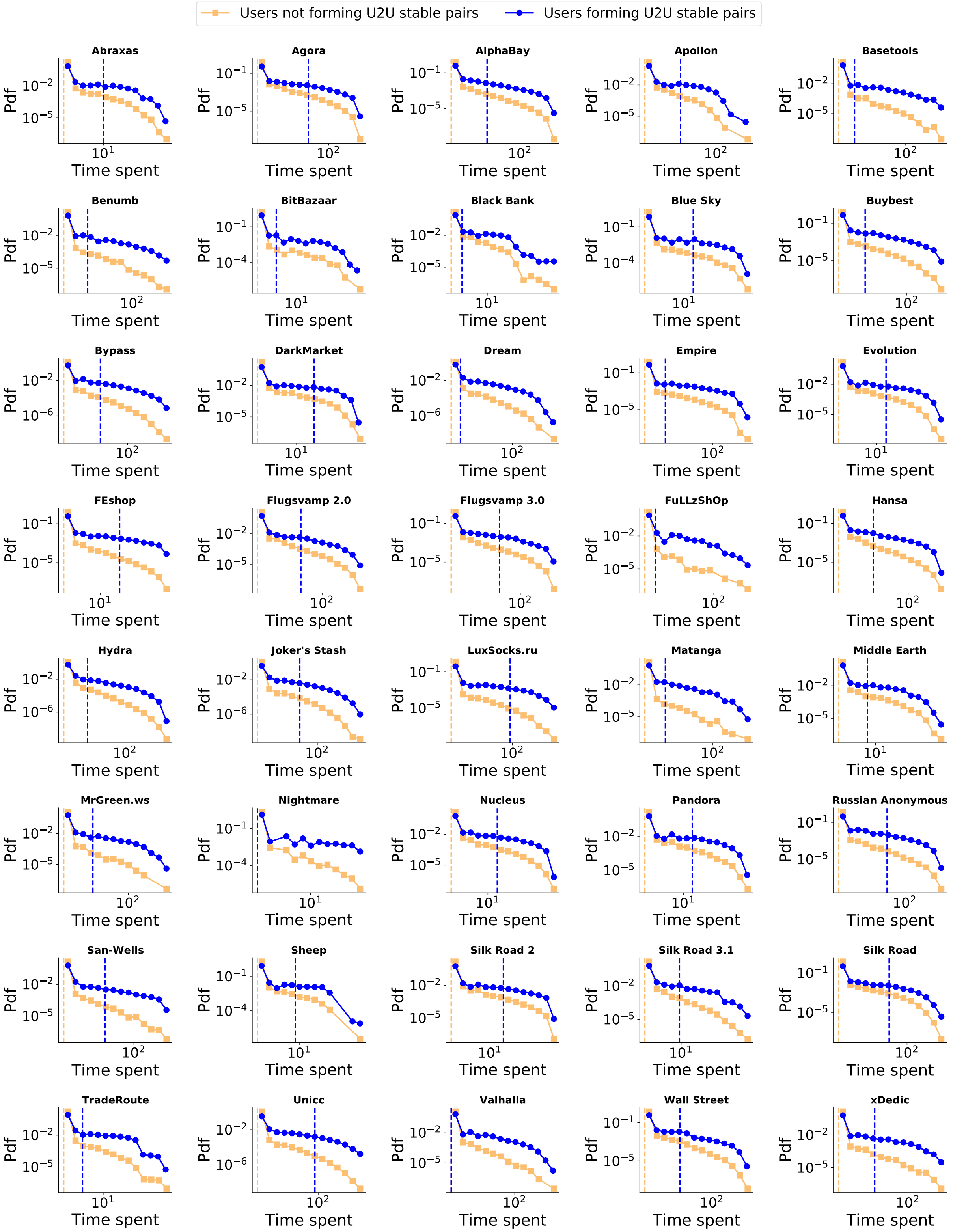

Figure S5. Evaluation of the time users spent on a DWM. It extends Figure 3(inset) in the main text by considering each individual DWM. Statistical tests are carried using the two-sided Kolmogorov-Smirnov test and results are available in Table S3. Vertical lines represent median values of the respective distributions.

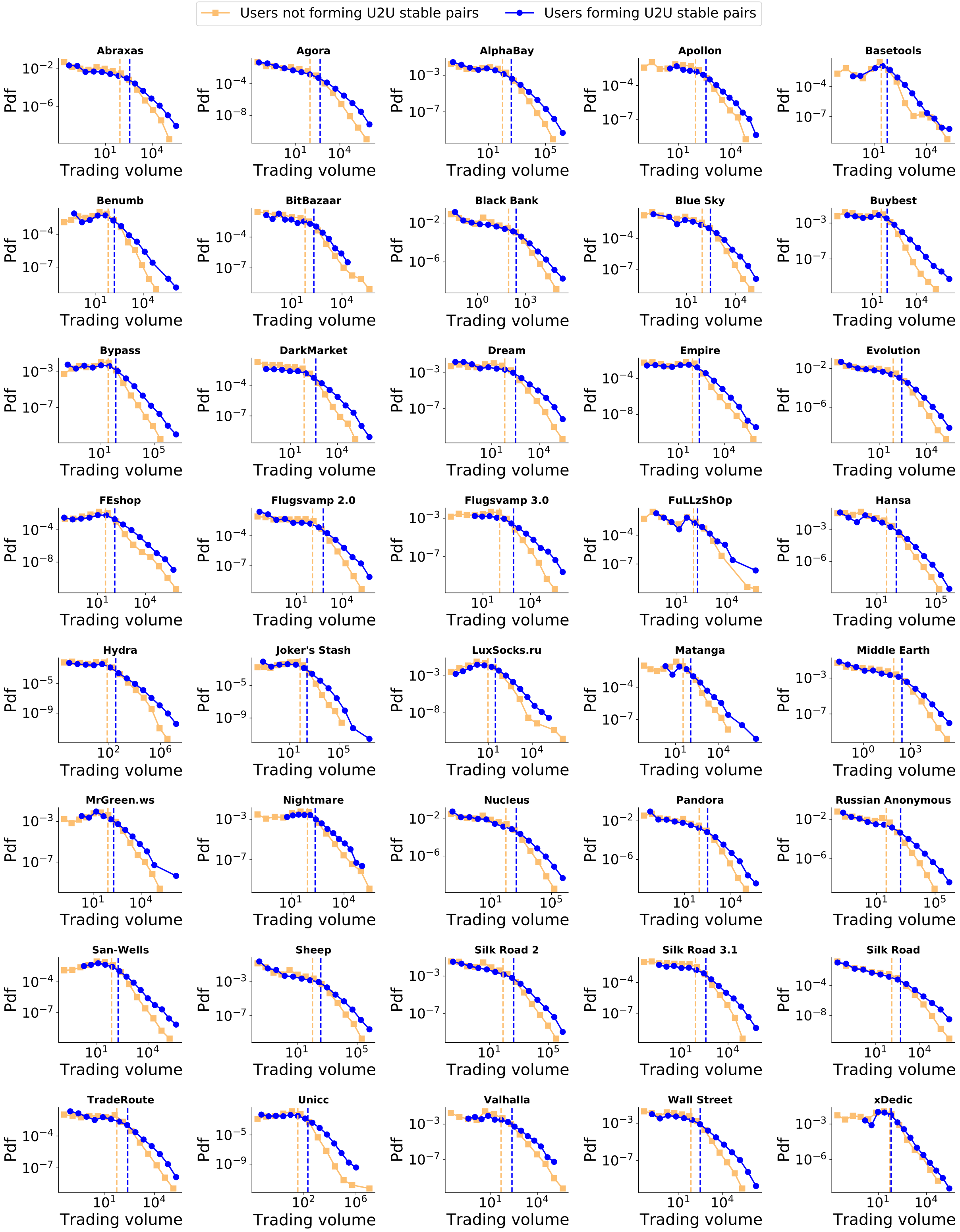

Figure S6. Evaluation of the total trading volume users exchange with a DWM. It extends Figure 3 in the main text by considering each individual DWM. Statistical tests are carried using the two-sided Kolmogorov-Smirnov test and results are available in Table S3. Vertical lines represent median values of the respective distributions.

Name	Time spent on a DWM	Trading volume exchanged with a DWM
	Users with stable U2U pairs vs other users (KS; p-value)	Users with stable U2U pairs vs other users (KS; p-value)
Abraxas	(0.529; 0.0001)	(0.355; 0.0001)
Agora	(0.583; 0.0001)	(0.351; 0.0001)
AlphaBay	(0.561; 0.0001)	(0.327; 0.0001)
Apollon	(0.540; 0.0001)	(0.382; 0.0001)
Basetools	(0.504; 0.0001)	(0.394; 0.0001)
Benumb	(0.519; 0.0001)	(0.286; 0.0001)
BitBazaar	(0.482; 0.0001)	(0.394; 0.0001)
Black Bank	(0.409; 0.0001)	(0.290; 0.0001)
Blue Sky	(0.535; 0.0001)	(0.346; 0.0001)
Buybest	(0.534; 0.0001)	(0.338; 0.0001)
Bypass	(0.584; 0.0001)	(0.426; 0.0001)
DarkMarket	(0.635; 0.0001)	(0.459; 0.0001)
Dream	(0.475; 0.0001)	(0.450; 0.0001)
Empire	(0.491; 0.0001)	(0.395; 0.0001)
Evolution	(0.557; 0.0001)	(0.305; 0.0001)
FEshop	(0.639; 0.0001)	(0.447; 0.0001)
Flugsvamp 2.0	(0.551; 0.0001)	(0.423; 0.0001)
Flugsvamp 3.0	(0.604; 0.0001)	(0.500; 0.0001)
FuLLzShOp	(0.513; 0.0001)	(0.222; 0.0001)
Hansa	(0.554; 0.0001)	(0.359; 0.0001)
Hydra	(0.536; 0.0001)	(0.327; 0.0001)
Joker’s Stash	(0.647; 0.0001)	(0.389; 0.0001)
LuxSocks.ru	(0.676; 0.0001)	(0.390; 0.0001)
Matanga	(0.544; 0.0001)	(0.405; 0.0001)
Middle Earth	(0.473; 0.0001)	(0.317; 0.0001)
MrGreen.ws	(0.537; 0.0001)	(0.280; 0.0001)
Nightmare	(0.401; 0.0001)	(0.400; 0.0001)
Nucleus	(0.559; 0.0001)	(0.380; 0.0001)
Pandora	(0.505; 0.0001)	(0.313; 0.0001)
Russian Anonymous	(0.682; 0.0001)	(0.533; 0.0001)
San-Wells	(0.558; 0.0001)	(0.302; 0.0001)
Sheep	(0.472; 0.0001)	(0.344; 0.0001)
Silk Road 2	(0.539; 0.0001)	(0.365; 0.0001)
Silk Road 3.1	(0.542; 0.0001)	(0.415; 0.0001)
Silk Road	(0.589; 0.0001)	(0.341; 0.0001)
TradeRoute	(0.496; 0.0001)	(0.386; 0.0001)
Unicc	(0.708; 0.0001)	(0.537; 0.0001)
Valhalla	(0.448; 0.0001)	(0.440; 0.0001)
Wall Street	(0.562; 0.0001)	(0.405; 0.0001)
xDedic	(0.546; 0.0001)	(0.172; 0.0001)

Table S3. Statistical tests. The two-sided Kolmogorov-Smirnov test is used to perform the statistical test. All p-values are less than 0.0001, which is indicated with 0.0001.

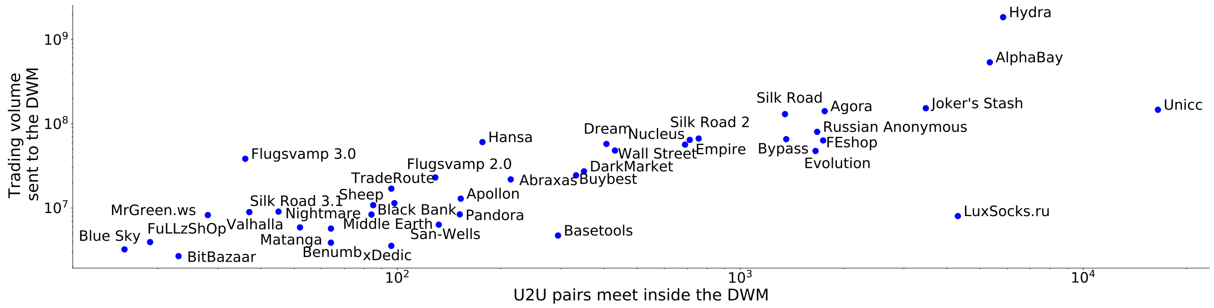

Figure S7. DWMs where users meet. Scatter plot of the number of pairs of users that meet inside each of the 40 DWMs considered versus the total volume sent to the DWM.

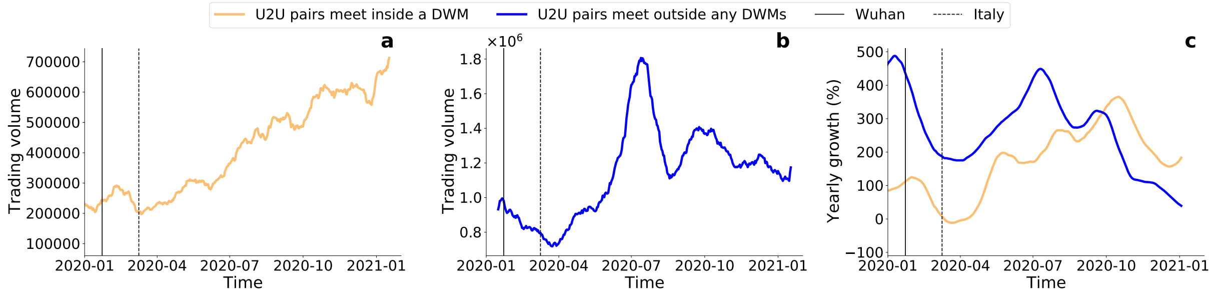

Figure S8. Trading volume stable U2U pairs during COVID-19. 28-days moving average of trading volume between users who met inside a DWM (a) and outside any DWMs (b). (c) Yearly growth relative to the same day of 2019. Vertical lines represent the dates of Wuhan (Jan 23, 2020) and Italy (March 3, 2020) lockdowns.

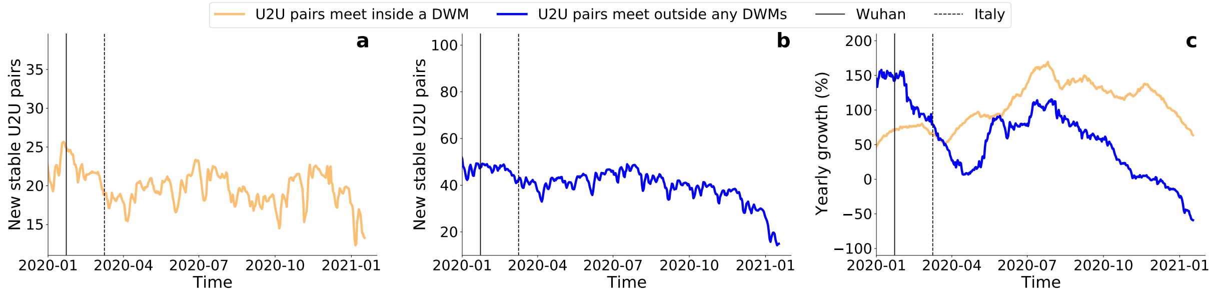

Figure S9. Formation of new stable U2U pairs during COVID-19. 28-days moving average of new stable U2U pairs started between U2U pairs who met inside a DWM (a) and outside any DWMs (b). (c) Yearly growth relative to the same day of 2019. Vertical lines represent the dates of Wuhan and Italy lockdowns.

References

1. Chainalysis. The Chainalysis 2021 crypto crime report. https://go.chainalysis.com/2021-Crypto-Crime-Report.html Accessed October 27, 2021 (2021).
2. Chainalysis. The 2021 global crypto adoption index: Worldwide adoption jumps over 880% with p2p platforms driving cryptocurrency usage in emerging markets. https://blog.chainalysis.com/reports/2021-global-crypto-adoption-index Accessed October 27, 2021 (2020).
3. Khalilov, M. C. K. & Levi, A. A survey on anonymity and privacy in Bitcoin-like digital cash systems. *IEEE Commun. Surv. & Tutorials* **20**, 2543–2585 (2018).

4. Wilcoxon, F. Individual Comparisons by Ranking Methods. In *Breakthroughs in Statistics*, 196–202 (Springer, 1992).

5. Mann, H. B. & Whitney, D. R. On a test of whether one of two random variables is stochastically larger than the other. *The Annals Math. Stat.* 50–60 (1947).

6. Massey Jr, F. J. The Kolmogorov-Smirnov test for goodness of fit. *J. Am. statistical Assoc.* **46**, 68–78 (1951).

7. Spearman, C. The proof and measurement of association between two things. *Appleton-Century-Crofts* (1961).

8. Darknetlive. https://darknetlive.com/markets/darkbay/ Accessed October 27, 2021 (2020).

9. Cuthbertson, A. Coronavirus: dark web market bans drug dealers selling fake COVID-19 vaccines. https://www.independent.co.uk/life-style/gadgets-and-tech/news/coronavirus-vaccine-cure-dark-web-drugs-market-covid-19-a9442671.html Accessed October 27, 2021 (2020). Independent.

10. Wehinger, F. The dark net: Self-regulation dynamics of illegal online markets for identities and related services. In *2011 European Intelligence and Security Informatics Conference*, 209–213 (IEEE, 2011).

11. Soska, K. & Christin, N. Measuring the longitudinal evolution of the online anonymous marketplace ecosystem. In *24th {USENIX} security symposium ({USENIX} security 15)*, 33–48 (2015).

12. Emcdda special report: COVID-19 and drugs – drug supply via darknet markets. https://www.emcdda.europa.eu/publications/ad-hoc/covid-19-and-drugs-drug-supply-via-darknet-markets_en Accessed October 27, 2021 (2020). European Monitoring Centre for Drugs and Drug Addiction (EMCDDA).

13. Bracci, A. *et al.* Dark Web Marketplaces and COVID-19: Before the vaccine. *EPJ Data Sci.* **10** (2021).

14. Dread forum. https://onion.live/site/dread-forum Accessed via Tor browser October 27, 2021 (2020).

15. Raptor.life. Your most trusted darknet markets links directory. https://raptor.life/index.php Accessed October 27, 2021 (2020).

16. Darknetlive. https://darknetlive.com/ Accessed October 27, 2021 (2020).

17. Dark.fail. https://dark.fail/ Accessed October 27, 2021 (2020).

18. Barratt, M. J., Ferris, J. A. & Winstock, A. R. Use of Silk Road, the online drug marketplace, in the United Kingdom, Australia and the United States. *Addiction* **109**, 774–783 (2014).

19. Martin, J. Lost on the Silk Road: Online drug distribution and the cryptomarket. *Criminol. & Crim. Justice* **14**, 351–367 (2014).

20. Aldridge, J. & Décary-Hétu, D. Not an Ebay for drugs: The cryptomarket Silk Road as a paradigm shifting criminal innovation. *Available at SSRN 2436643* (2014).

21. Martin, J. *Drugs on the dark net: How cryptomarkets are transforming the global trade in illicit drugs* (Springer, London, UK, 2014).

22. Dingledine, R., Mathewson, N. & Syverson, P. Tor: The second-generation onion router. Tech. Rep., Naval Research Lab Washington DC (2004).

23. Nakamoto, S. Bitcoin: A peer-to-peer electronic cash system. Tech. Rep., Manubot (2008).

24. Lee, S. *et al.* Cybercriminal minds: An investigative study of cryptocurrency abuses in the dark web. In *Network and Distributed System Security Symposium*, 1–15 (Internet Society, 2019).

25. Foley, S., Karlsen, J. R. & Putniņš, T. J. Sex, drugs, and Bitcoin: How much illegal activity is financed through cryptocurrencies? *The Rev. Financial Stud.* **32**, 1798–1853 (2019).

26. Möser, M. *et al.* An empirical analysis of traceability in the monero blockchain. *Proc. on Priv. Enhancing Technol.* **2018**, 143–163 (2018).

27. Bitcoincore. https://bitcoin.org/en/bitcoin-core/ Accessed October 27, 2021 (2020).

28. Blockchain.com. www.blockchain.com Accessed October 27, 2021 (2020). Daily Mail.

29. Ron, D. & Shamir, A. Quantitative analysis of the full Bitcoin transaction graph. In *International Conference on Financial Cryptography and Data Security*, 6–24 (Springer, 2013).

30. Androulaki, E., Karame, G. O., Roeschlin, M., Scherer, T. & Capkun, S. Evaluating user privacy in Bitcoin. In *International Conference on Financial Cryptography and Data Security*, 34–51 (Springer, 2013).

31. Tasca, P., Hayes, A. & Liu, S. The evolution of the Bitcoin economy. *The J. Risk Finance* (2018).

32. Harrigan, M. & Fretter, C. The unreasonable effectiveness of address clustering. In *2016 Intl IEEE Conferences on Ubiquitous Intelligence & Computing, Advanced and Trusted Computing, Scalable Computing and Communications, Cloud and Big Data Computing, Internet of People, and Smart World Congress (UIC/ATC/ScalCom/CBDCom/IoP/SmartWorld)*, 368–373 (IEEE, 2016).

33. Meiklejohn, S. *et al.* A fistful of Bitcoins: Characterizing payments among men with no names. In *Proceedings of the 2013 conference on Internet measurement conference*, 127–140 (2013).

34. Wikipedia now accepts bitcoin donations. https://www.coindesk.com/wikipedia-now-accepts-bitcoin-donations Accessed October 27, 2021 (2014). Coindesk.

35. Chainalysis, inc. https://www.chainalysis.com/ Accessed October 27, 2021 (2020).

36. Chung, Y. Cracking the code: How the us government tracks Bitcoin transactions. *Analysis Of Appl. Math.* 152 (2019).

37. Chainalysis. Chainalysis in action: How law enforcement used blockchain analysis to follow funds and identify the twitter hackers. https://blog.chainalysis.com/reports/chainalysis-doj-twitter-hack-2020 Accessed October 27, 2021 (2020).

38. Nadini, M., Bongiorno, C., Rizzo, A. & Porfiri, M. Detecting network backbones against time variations in node properties. *Nonlinear Dyn.* **99**, 855–878 (2020).

39. Scargle, J. D., Norris, J. P., Jackson, B. & Chiang, J. Studies in astronomical time series analysis. VI. Bayesian block representations. *The Astrophys. J.* **764**, 167 (2013).
